# Assessment of the Provider Relief Fund Distribution for Treatment of Uninsured Patients With COVID-19

**DOI:** 10.1001/jamahealthforum.2021.4695

**Published:** 2022-01-28

**Authors:** Stephen T. Parente, Karoline Mortensen

**Affiliations:** 1 Carlson School of Management, University of Minnesota, Minneapolis; 2 Herbert Business School, University of Miami, Coral Gables, Florida

## Abstract

This cross-sectional study uses US Health Resources and Services Administration data to assess the distribution of claims reimbursement funds to health care professionals and facilities for uninsured patients with COVID-19.

## Introduction

The Health Resources and Services Administration (HRSA) within the US Department of Health and Human Services (HHS) began covering COVID-19 related testing and treatment costs for those without health insurance during the COVID-19 pandemic in April 2020.^1,2,3,4^ The Provider Relief Fund (PRF) operates a Medicare-like fee-for-service claims reimbursement system, reimbursing health care clinicians and facilities for each uninsured claim at the Medicare payment rate. As of September 2021, $2.5 billion had been paid by HRSA for COVID-19 treatment of uninsured patients with COVID-19.^5^ A policy choice was made to create the PRF rather than implement an emergency expansion of Medicaid or a special marketplace enrollment period. To our knowledge, there has been no analysis of the distribution of funds to health care professionals and facilities treating uninsured patients. This study aims to evaluate this method of distribution of disbursement relative to the number of uninsured people in a state.

## Methods

We computed state-level estimates of per capita uninsured COVID-19 treatment reimbursements and compared the distribution of COVID-19 PRF funds to the percent of the US uninsured population (aged 19-64 years) in each state. This cross-section analysis used publicly available HRSA claims data detailing federal PRF reimbursement for treatment of uninsured patients^5^ and data on the distribution of the US uninsured population in 2020.^6^ This cross-sectional study followed Strengthening the Reporting of Observational Studies in Epidemiology (STROBE) reporting guideline. Because the data are administrative and have no patient identifying information, this study did not meet the definition of human particpants research as defined by the Common Rule (45 CFR §46) and was not reviewed by an institutional review board.

We focused on state-level data because of differential access to insurance due to states’ decisions to offer coverage via the Affordable Care Act Medicaid expansion. States have been classified as either a Medicaid expansion state or a non-Medicaid expansion state. We constructed a reimbursement ratio for each state in which the numerator is the state’s share of national PRF COVID-19 reimbursement and the denominator is the state’s share of uninsured patients in the US. A number above 1 is interpreted as a greater share of the PRF funds to a state than the state’s share of the uninsured population. A reimbursement ratio less than 1 indicates less treatment reimbursement to a state relative to its share of the uninsured population. Analyses were conducted using Microsoft Excel, version 16.50 (Microsoft Corporation).

## Results

The variance in distribution of the US uninsured population and the distribution of treatment funds to clinicians and facilities treating uninsured patients with COVID-19 suggests that some states received greater PRF shares than their share of uninsured patients. The Figure shows the reimbursement ratio of national share of PRF received by state to national share of the uninsured population by state in 12 non-Medicaid expansion states (24%) and 38 Medicaid expansion states (76%). Among the states not expanding Medicaid, 4 of 12 states (33%) had reimbursement ratios above 1.00 (Texas, 1.78; Georgia, 1.33; Tennessee, 1.12; and North Carolina, 1.07). Among the Medicaid expansion states, only 3 of 38 states (8%) had reimbursement ratios above 1.00 (New Jersey, 2.63; Maryland, 1.55; and Nevada, 1.01). In addition, the District of Columbia had a ratio of 4.25.

**Figure.**
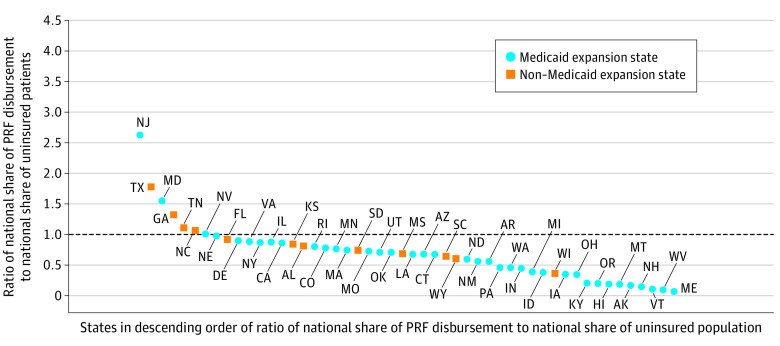
Reimbursement Ratio of National Share of PRF Disbursement Received by State to National Share of Uninsured by State Calculated from public claims data available through the US Department of Health and Human Services.^5^ PRF indicates Provider Relief Fund of the US Department of Health and Human Services.

## Discussion

The results suggest that states with greater than the national share of uninsured patients received greater shares of COVID-19 uninsured treatment funding. A limitation of this analysis is that billing the professional or facility location may not reflect the actual health care facility or patient location. The health care professional or facility location is a function of entity size and corporate strategy. However, we assume that small practices have co-located billing departments and that larger hospitals have regional corporate headquarters located in a specific state due to regulatory requirements.

There are 3 important policy implications. First, states with the largest uninsured populations such as Texas, California, and Florida were among those with the highest reimbursement ratios, suggesting that the states with the greatest population demands received the greatest share of funds. Second, Medicaid expansion states generally used less than their proportionate share of the program distribution. However, 3 states with ratios greater than 1.0 were Medicaid expansion states. In addition, as a policy mechanism to address the largest national health crisis in generations, the program served rough justice (a set of quick and decisive actions by the HHS leadership without prior policy precedent in a time of national crisis) to states with some of the highest shares of the uninsured population. The reasons the reimbursement ratio varied so much among Medicaid expansion states might be a useful topic for future research to help illuminate potential responses to a public health emergency.
